# The status and challenges of optogenetic tools for precise spatiotemporal control of RNA metabolism and function

**DOI:** 10.1002/ctm2.1078

**Published:** 2022-10-17

**Authors:** Renmei Liu, Mengyue Fang, Xianjun Chen, Yi Yang

**Affiliations:** ^1^ Optogenetics & Synthetic Biology Interdisciplinary Research Center State Key Laboratory of Bioreactor Engineering Shanghai Frontiers Science Center of Optogenetic Techniques for Cell Metabolism East China University of Science and Technology Shanghai China; ^2^ School of Pharmacy East China University of Science and Technology Shanghai China

1

RNA is the cornerstone of biology's central dogma, which was initially thought to serve only as an intermediate between DNA and protein. Decades of research, in particular the discovery of new classes of non‐coding RNAs (ncRNAs), have unveiled a plethora of activities that RNAs can fulfil besides coding for proteins, ranging from catalysis over scaffolding to regulatory functions. These ncRNAs not only play important roles in healthy individuals, but also are implicated in a wide range of diseases, including cancers, cardiovascular and neurological diseases, which have been demonstrated in several clinical studies.^1^ For this reason, the study of RNA metabolism, including transcription, pre‐mRNA processing, mRNA export, RNA trafficking and translation, represents a crucial milestone for understanding the biology of cells and molecular pathology of disease. However, when compared to our knowledge of proteins and genomes, our understanding of RNA's diverse biological roles is significantly lacking, in part because of the transient and complex dynamics of RNA and the challenges associated with precisely manipulating RNA metabolism from synthesis to degradation. While methods so far developed have made a significant impact in shedding light on the mysteries of RNA, tools that allow precise spatiotemporal control of RNA metabolism are still urgently needed for deeper insight into the diverse physiological functions of RNA.

Compared to classical genetic or pharmacologic perturbations, light is a very attractive actuator that can be used to manipulate cellular function and intracellular signalling pathways with millisecond and submicron resolutions.^2^ The use of light to control heterologously expressed proteins that are designed to be light responsive is generally termed optogenetics. Previously, a number of optogenetic systems based on the light‐induced dimerization or signalling cascade have been developed to allow spatiotemporal control of RNA synthesis and gene expression in diverse organisms.^3^ These light‐controlled gene transcription systems provide valuable tools for a variety of biotechnological and biomedical applications, ranging from in vitro basic research to in vivo gene‐ and cell‐based therapies.

Unlike the extensive studies on developing optogenetic tools for transcriptional control, reports on optogenetic control of post‐transcriptional RNA metabolism are much fewer. Cao et al. developed optogenetic systems that can activate mRNA translation in mammalian cells in a light‐dependent manner. In these systems, blue light induces the reconstitution of an RNA binding domain and a translation initiation domain, thereby activating the translation of target mRNA downstream of the binding sites.^4^, ^5^ Moreover, Kim et al. engineered the mRNA LARIAT (mRNA light‐activated reversible inactivation by assembled trap) system, in which the LARIAT system is combined with RNA‐binding protein (RBP)‐based mRNA visualization modules to trap specific mRNAs in protein clusters using light.^6^ Such sequestration of mRNAs can restrict their accessibility to ribosomes and thus markedly reduce translation efficiency. The mRNA LARIAT system allows precise manipulation of the localization and translation of both exogenous and endogenous unmodified mRNAs. While as promising as they appear to be, uptake of these tools by biologists has been minimal, probably because of technical complexities or limitations.

In eukaryotic cells, RBPs are key components in RNA metabolism, regulating the temporal, spatial and functional dynamics of RNAs. Thus, the engineering of light‐switchable RBPs would provide an easier and more straightforward way to control the post‐transcriptional metabolism of RNA. Weber et al. selected an RNA aptamer that can specifically bind a natural blue light receptor, termed PAL, in a light‐dependent manner. PAL allows optogenetic control of mRNA translation both in bacteria and mammalian cells.^7^ Very recently, Liu et al. engineered the LicV, a synthetic photoswitchable RBP that binds to a specific RNA sequence in response to light irradiation.^8^ Blue light can induce the dimerization of LicV and enhance its binding to the RAT RNA sequence, and removal of the light results in gradual dissociation of the dimers and RNA dissociation. As a consequence, upon blue light exposure, LicV binds RAT with a low dissociation constant (*K*_d_) of 92 nM, but in the dark, the binding is >40‐fold weaker with a *K*_d_ of 3793 nM. LicV can serve as an excellent scaffold for the development of photoswitchable RNA effectors that allow spatiotemporal control of the localization, splicing, translation and stability of the target RNAs, by simplify fusing LicV to RNA effectors with the corresponding functionalities. Furthermore, LicV‐based RNA effectors can be combined with CRISPR‐Cas system for highly efficient and tunable regulation of RNA synthesis from both exogenous and endogenous genes (see Figure 1). Given the favourable photoinducible properties of the LicV, the LicV‐based optogenetic tools will provide particularly attractive ways for spatiotemporal control of post‐transcriptional RNA metabolism.

**FIGURE 1:**
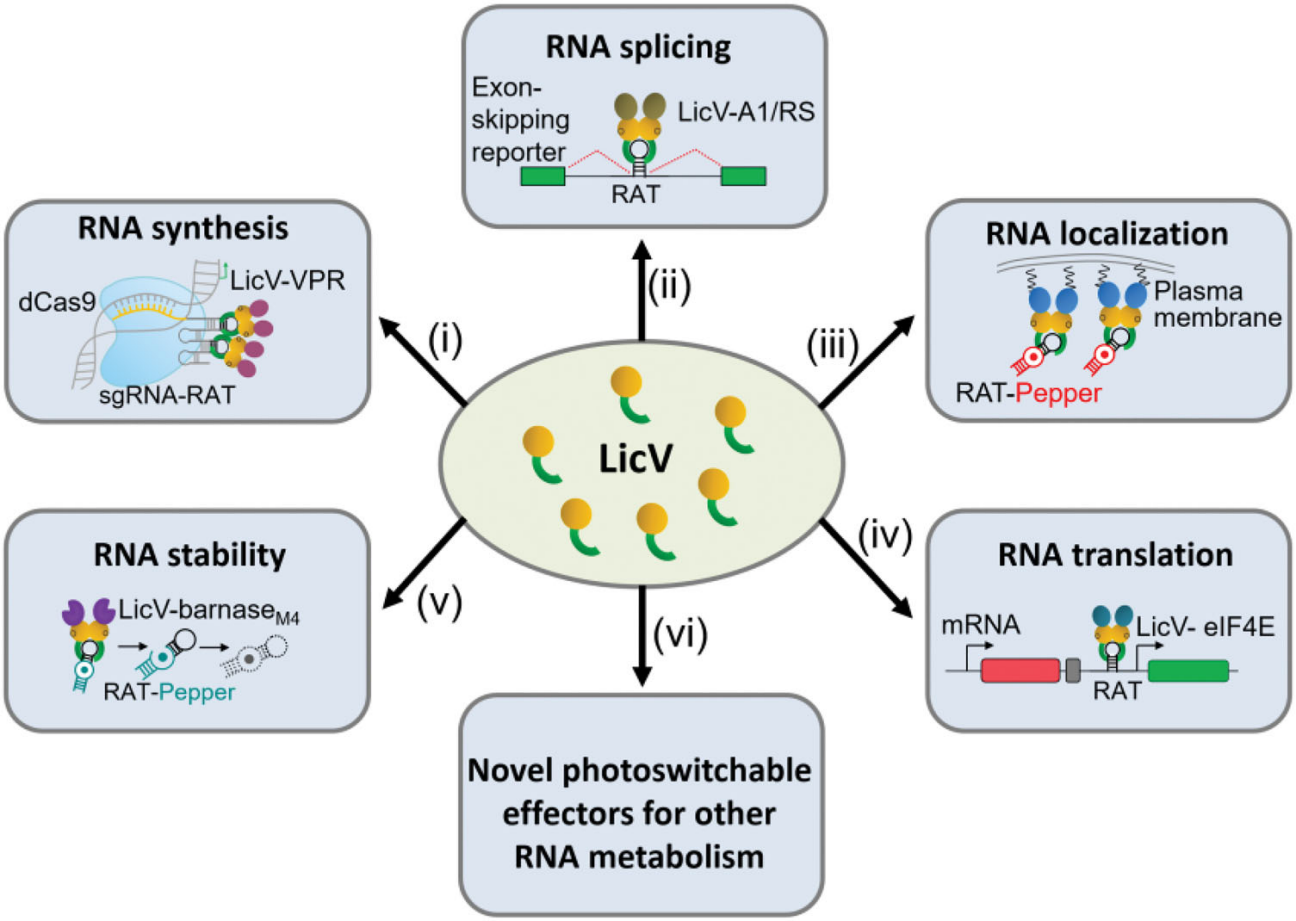
LicV‐based photoswitchable effectors for spatiotemporal control of RNA metabolism. The synthetic photoswitchable RNA binding protein LicV is fused to various functional domains, including transcriptional activation domain (i), splicing modulatory domain (ii), subcellular localization signal (iii), translation initiation factor (iv), endonuclease domain (v) and other effector domains (vi), for spatiotemporal control of RNA synthesis, splicing, localization, translation, stability and other RNA metabolism, respectively

Today, scientists’ interests in the contribution of RNA to the genesis and progression of human disorders are booming, but the functions of the vast majority of ncRNAs are still mysterious, and further studies are highly warranted to uncover their biological roles and the mechanisms by which ncRNAs exert their pathological effects. Light may be useful for precisely controlling the expression levels or subcellular localization of RNAs, thereby enabling scientists to link changes in cellular functions with changes in the intracellular availability of these RNAs. Despite the significant progress in developing RNA optogenetic tools, significant challenges still need to be overcome to expand the toolbox of RNA optogenetic tools for unlocking the deeper mysteries of RNA. First, previously developed optogenetic tools permit spatiotemporal control of a single RNA each time. However, many biological processes require the concerted action of multiple RNAs, for example, RNA splicing and ribosome assembly. To enable the precise control of these processes, it is necessary to engineer orthogonal and multichromatic optogenetic tools for simultaneous control of multiple RNAs. Second, most of the existing RNA optogenetic tools utilize blue light as the actuator, engineering of optogenetic tools that respond to red or infrared light would be more relevant for triggering RNA molecules in vivo, as light in this range is less toxic to cells and can penetrate tissues more efficiently than blue light. Third, optogenetic tools for the control of RNA epigenetics may also be engineered by fusing LicV to write or erase the functionalities of RNA methylation, as well as control of site‐directed RNA editing by fusing LicV to the deaminase domains. The combination of these optogenetic tools will provide an opportunity to control the functions of different RNAs differentially and orthogonally, which may be particularly useful for the study of RNAs with antagonistic or synergetic functions.

## CONFLICT OF INTEREST

All the authors declare that there is no potential conflict of interest.

## References

[1] Esteller M . Non‐coding RNAs in human disease. Nat Rev Genet. 2011;12:861‐874. 22094949 10.1038/nrg3074

[2] Gautier A , Gauron C , Volovitch M , Bensimon D , Jullien L , Vriz S . How to control proteins with light in living systems. Nat Chem Biol. 2014;10:533‐541. 24937071 10.1038/nchembio.1534

[3] Mansouri M , Strittmatter T , Fussenegger M , Light‐controlled mammalian cells and their therapeutic applications in synthetic biology. Adv Sci 2019; 6:1800952. 10.1002/advs.201800952 PMC6325585 30643713

[4] Cao J , Arha M , Sudrik C , Bugaj LJ , Schaffer DV , Kane RS . Light‐inducible activation of target mRNA translation in mammalian cells. Chem Commun. 2013;49:8338‐8340. 10.1039/c3cc44866e 23925486

[5] Cao J , Arha M , Sudrik C , Schaffer DV , Kane RS . Bidirectional regulation of mRNA translation in mammalian cells by using PUF domains. Ang Chem. 2014;53:4900‐4904. 10.1002/anie.201402095 24677733

[6] Kim NY , Lee S , Yu J , et al. Optogenetic control of mRNA localization and translation in live cells. Nat Cell Biol. 2020;22:341‐352. 32066905 10.1038/s41556-020-0468-1

[7] Weber AM , Kaiser J , Ziegler T , et al. A blue light receptor that mediates RNA binding and translational regulation. Nat Chem Biol. 2019;15:1085‐1092. 31451761 10.1038/s41589-019-0346-y PMC6811359

[8] Liu R , Yang J , Yao J , et al. Optogenetic control of RNA function and metabolism using engineered light‐switchable RNA‐binding proteins. Nat Biotechnol. 2022;40:779‐786. 34980910 10.1038/s41587-021-01112-1

